# Laparoscopic infrapyloric lymph nodes dissection through the right bursa omentalis approach for gastric cancer

**DOI:** 10.1186/s12893-021-01192-5

**Published:** 2021-04-26

**Authors:** Kun Yang, Wei-Han Zhang, Kai Liu, Xin-Zu Chen, Xiao-Long Chen, Zong-Guang Zhou, Jian-Kun Hu

**Affiliations:** 1 Department of Gastrointestinal Surgery, West China Hospital, Sichuan University, No. 37 Guo Xue Xiang Street, Chengdu, 610041 Sichuan Province China; 2 Institute of Gastric Cancer, State Key Laboratory of Biotherapy/Collaborative Innovation Center of Biotherapy and Cancer Center, West China Hospital, Sichuan University, Chengdu, China; 3 Department of Gastrointestinal Surgery, West China Hospital Sichuan Univerity Jintang Hospital, Chengdu, China

**Keywords:** Gastric cancer, Laparoscopic gastrectomy, Infrapyloric lymph nodes, No.6 lymph nodes, Bursa omentalis

## Abstract

**Background:**

A complete dissection of infrapyloric lymph nodes is the key to a curative gastrectomy, which can be sometimes technically challenging in laparoscopic surgery.

**Methods:**

One hundred and eighteen patients with gastric cancer undergoing laparoscopic gastrectomy with D2 lymphadenectomy in which the infrapyloric lymph nodes were dissected through the right bursa omentalis approach were included. The clinicopathologic characteristics and surgical outcomes were analyzed retrospectively.

**Results:**

The laparoscopic gastrectomy with D2 lymphadenectomy was successful in all 118 patients with no open conversion. The mean operation time was 246.6 ± 45.7 min. The mean estimated blood loss was 87.0 ± 35.9 mL. Postoperative complications occurred in 17.8% of the patients, which were treated successfully with conservative therapy or aspiration in all. There were no No.6 lymphadenectomy-associated complications, such as injury of transverse colon, vessels of mesocolon, pancreas or duodenum, no pancreatitis, pancreatic leakage or postoperative hemorrhage. The mean postoperative hospital stay was 9.6 ± 3.7 days. On average, the total lymph nodes harvested were 36.8 ± 12.9, in which the ones from the infrapyloric area were 5.1 ± 3.1.

**Conclusion:**

Laparoscopic dissection of infrapyloric lymph nodes through the right bursa omentalis approach seems to be feasible and safe, facilitating a more complete No.6 lymphadenectomy for gastric cancer.

**Supplementary Information:**

The online version contains supplementary material available at 10.1186/s12893-021-01192-5.

## Introduction

The prevalence of gastric cancer has decreased worldwide in the past 20 years. However, it still remains a malignant disease with comparably high incidence and mortality especially in East Asia [1–3]. Distal gastric cancer remains the most common type, although in recent years the incidence of proximal gastric cancer has gradually increased [4, 5]. Surgery is still the cornerstone of a comprehensive treatment for gastric cancer in which a radical lymphadenectomy is the key [6]. Nowadays, D2 lymphadenectomy has been widely accepted as a standard procedure since it has been proven effective to reduce the risk of tumor recurrence and improve the overall survival of patients with gastric cancer [7].

The No.6 lymph nodes are defined as the infrapyloric lymph nodes along the first branch and proximal part of the right gastroepiploic artery down to the confluence of the right gastroepiploic vein and the anterior superior pancreatoduodenal vein [8]. The No.6 lymph nodes have been reported of high frequency of metastasis in gastric cancer (up to approximately 40% in advanced cases, [9] especially the tumor in the distal 1/3 of the stomach). In the Japanese Gastric Cancer Treatment Guideline, it is recommended that the dissection of No.6 lymph nodes needs to be complete, even in the extent of D1 lymphadenectomy for distal and total gastrectomy [6]. Therefore, a thorough and precise dissection of the No.6 lymph nodes is critical.

Laparoscopic gastrectomy, the minimal invasive approach, has been increasingly used to treat gastric cancer. In the Japanese Gastric Cancer Treatment Guidelines, laparoscopic distal gastrectomy has been recommended as a treatment option for early gastric cancers [6]. Meanwhile, laparoscopic distal gastrectomy for advanced cancers and laparoscopic total gastrectomy have also been reported of comparable short-term and long-term outcomes to open surgery [10–13]. Nevertheless, accomplishing a complete and safe laparoscopic dissection of the No.6 lymph nodes is sometimes technically challenging, due to not only an intricate network and multiple anatomical variations of blood vessels in the infrapyloric area, but also the adjacent organs such as pancreas and transverse mesocolon which increase the technical difficulty and are vulnerable to potential intra-procedural injury [14]. Therefore, defining a proper surgical dissection plane with an appropriate technique is essential for a successful laparoscopic dissection of No.6 lymph nodes.

In order to facilitate a complete and safe lymphadenectomy, we have proposed a model called “clockwise modularized lymphadenectomy” for laparoscopic dissection of lymph nodes, and compared it with traditional laparoscopic lymphadenectomy [15]. In this article, we aimed to describe our surgical technique in detail for laparoscopic dissection of the infrapyloric lymph nodes through the right bursa omentalis approach, which is the important component of clockwise modularized lymphadenectomy, in order to cue a complete and safe laparoscopic No.6 lymphadenectomy and facilitate a wider application of this procedure. Additionally, we also sought to present the short-term outcomes of the procedure.

## Materials and methods

### Patients and indications

From January 2015 to July 2017, one hundred and eighteen patients with gastric cancer undergoing laparoscopic gastrectomy with D2 lymphadenectomy at West China Hospital, Sichuan University were included. Informed consent for operation was obtained preoperatively from all patients. The clinicopathologic characteristics and surgical outcomes, including demographic data, estimated blood loss, the number of harvested lymph nodes, TNM stage, perioperative and postoperative complications etc. were extracted from the prospective Surgical Gastric Cancer Patient Registry in West China Hospital. The application of data from this database has been approved by the Biomedical Ethics Committee of the West China Hospital, Sichuan University which also waived the patient informed consent due to the retrospective nature of the study [IRB No. 2014(215)]. All patients’ data were collected and analyzed anonymously.

All the patients included had been diagnosed of gastric cancer based on upper endoscopy and biopsy. And the preoperative contrasted abdominopelvic computed tomography was used for clinical staging. The indications for laparoscopic gastrectomy (together with curative D2 lymphadenectomy) were based on the Chinese laparoscopic gastrectomy guideline for gastric cancer (2016 edition), expert consensus on quality control of the laparoscopic radical resection for gastric cancer in China (2017 edition) and the inclusion and exclusion criteria of the CLASS-01 trials launched by the Chinese Laparoscopic Gastrointestinal Surgery Study (CLASS) group, [10, 16, 17] where the cancer stage was defined as cT1-3N0-2M0 at preoperative evaluation according to the 7th AJCC Cancer Staging [18] Additionally, the cT4a stage was considered as an explorative indication, should the expected curative D2 lymphadenectomy be achieved. Exclusion criteria were previous upper abdominal surgery (except laparoscopic cholecystectomy), eligibility for endoscopic treatments, and enlarged regional lymph node with the diameter larger than 3 cm.

### Surgical techniques

In the present study, we aimed to describe the novel surgical approach developed for infrapyloric lymph nodes dissection that has been gradually applied on all patients undergoing laparoscopic gastrectomy since 2014 at our institution. The aim of this delicate new approach was to reduce the iatrogenic injury of blood vessels and adjacent organs in a loose natural surgical plane, and eventually achieve a technically easier, safer and complete infrapyloric lymph nodes (No.6) dissection, rather than improve the survival through bursectomy. All the operations were performed by Jian-Kun Hu (operator), Kun Yang (assistant) and Xin-Zu Chen (assistant), who are highly experienced in this technique. The operation team has performed annually more than 200 gastrectomies with D2 lymphadenectomy with open and laparoscopic approaches and is certified by the CLASS academic committee (unedited operation Additional file 1: Video S1).

Under general anesthesia, the patient was placed in the supine position. The surgeon and camera holder stood on the patient’s right side and an assistant surgeon was on the patient’s left side. One 12 mm Trocar was placed just under the umbilicus to keep the pneumoperitoneal pressure at 12 ~ 14 mm Hg and function as the observation port. One 5 mm trocar was placed on the right anterior-axillary line just below the costal margin, and another 12-mm port was placed on the right mid-clavicular line 2–3 cm above the umbilicus. Two 5 mm trocars were placed on left anterior-axillary line below the costal margin and left mid-clavicular line 2–3 cm above the umbilicus respectively.

**Fig. 1:**
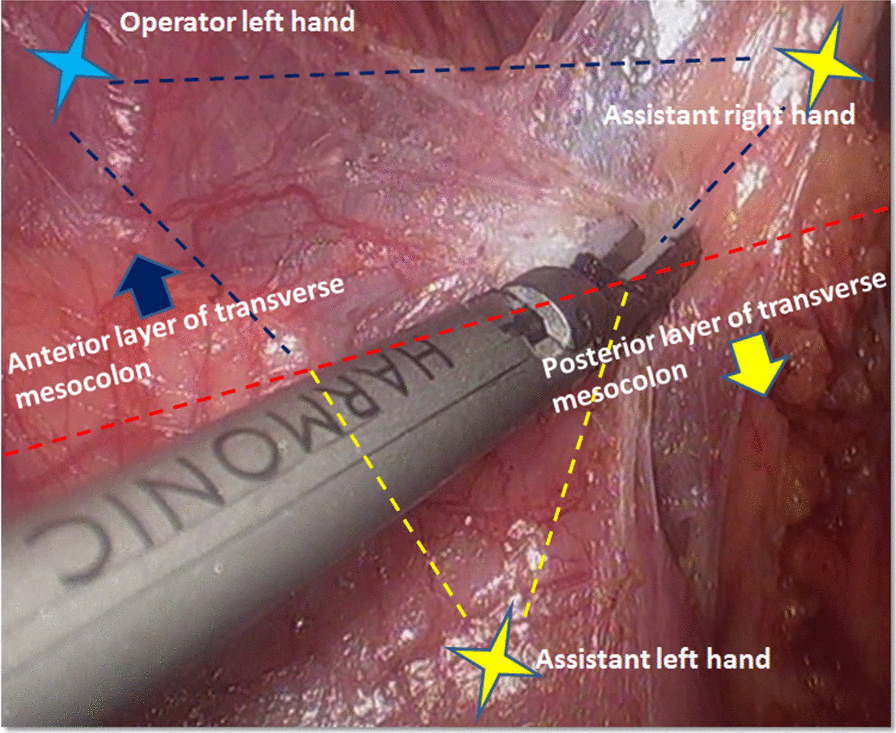
A “three-point technique” was adopted constantly to expose the interfascial space between the inner layer and external layer of omental bursa

The surgery began with liver retraction utilizing a purse-string suture. After the left-sided omentectomy and No.4sb lymph nodes dissection, the right-sided omentectomy was performed toward the hepatic flexure of colon. A “three point technique” was adopted constantly to expose the interfascial space between the inner layer and external layer of omental bursa. Ultrasonic scalpel was used for the dissection. The operator’s left hand and assistant’s right hand retracted and expanded the greater omentum (or anterior layer of transverse mesocolon), and the assistant’s left hand pulled the transverse colon caudally and dorsally. By retracting at 3 points, 2 planes were created by dissecting the loose interfascial space between the inner layer and external layer of the omental bursa. Thereafter the operator used the ultrasonic scalpel to dissect along the border shared between the two planes to separate the anterior layer of mesocolon (Fig. 1).

The operator’s left hand and the assistant’s right hand retracted and expanded the greater omentum, and the assistant’s left hand pulled the transverse colon caudally and dorsally and flattened the transverse mesocolon. The fourth layer of greater omentum was cut from the avascular area of the median part of gastrocolic ligament close to the transverse colon. Along this plane, the anterior layer of mesocolon peritoneum should be removed up to hepatic flexure and inferior border of pancreas cranially.

Since the interfascial space between the anterior layer and posterior layer of the mesocolon peritoneum is loose, a combination of blunt and sharp dissections can be adopted. The posterior layer can be pushed caudally with the ultrasonic scalpel. Blunt dissection of the anterior layer is also safe and very useful. When the anterior layer of transverse mesocolon peritoneum was dissected up to the inferior border of the pancreas, the surgical plane was re-oriented to the anterior surface of the pancreas. The ligaments between the duodenum and pancreas were dissected and the gastroduodenal artery was exposed, while the capsule of pancreas did not need to be peeled.

**Fig. 2:**
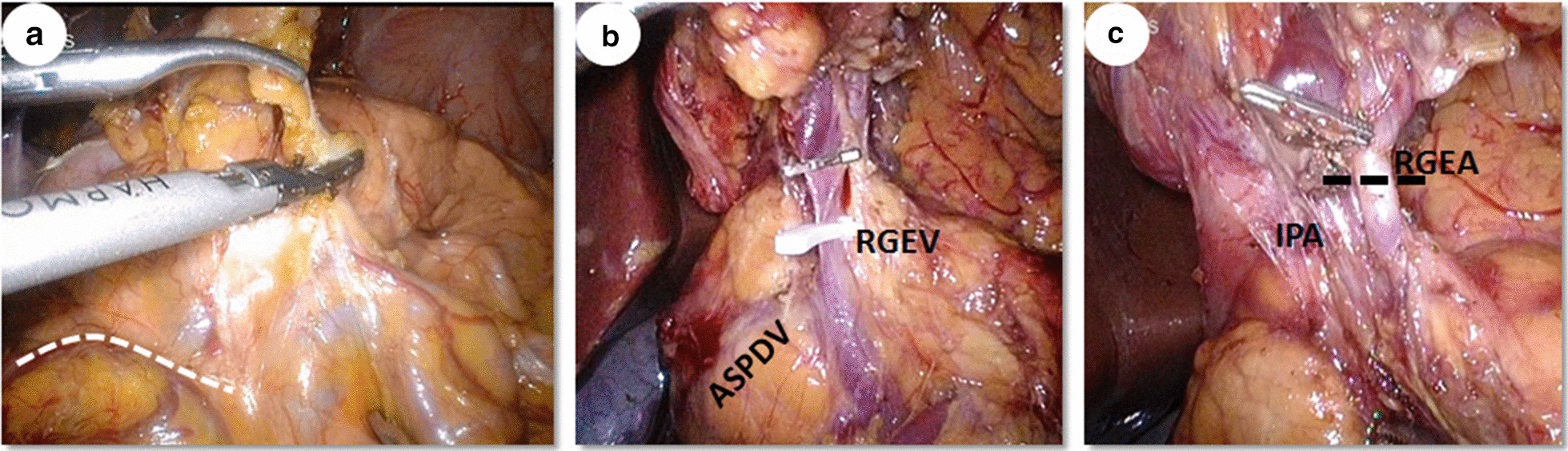
Laparoscopic infrapyloric lymph nodes dissection through the right bursa omentalis approach. **a** The groove between the head of pancreas and the transverse mesocolon should be exposed (indicated by the broken line). **b** The right gastroepiploic vein (RGEV) should be ligated and divided distal to the confluence point between the right gastroepiploic vein and anterior superior pancreaticoduodenal vein (ASPDV). **c** The right gastroepiploic artery (RGEA) should be divided just distal to the branching point of anterior superior pancreaticoduodenal artery from gastroduodenal artery (indicated by the broken line). *IPA* infrapyloric artery

For a complete No. 6 lymph nodes dissection, the groove between the head of pancreas and the transverse mesocolon should be exposed (Fig. 2a). Then, all the fatty and lymphatic tissues in front of the surface of the pancreatic head should be completely removed upward from the groove to duodenal bulb and leftward to the descending part of the duodenum. After dissecting this area, a continuous membrane extending from the posterior layer of transverse mesocolon to the pancreatic head can be seen. During the process, the confluence of anterior superior pancreaticoduodenal vein to the right gastroepiploic vein was displayed. And the right gastroepiploic vein should be ligated and divided distal to the confluence point between the right gastroepiploic vein and anterior superior pancreaticoduodenal vein (Fig. 2b). Afterwards, the No.6a lymph nodes surrounding the right gastroepiploic artery were dissected and the right gastroepiploic artery just distal to the branching point of anterior superior pancreaticoduodenal artery from gastroduodenal artery was divided (Fig. 2c). Attention was paid to the nearby infrapyloric artery that also needed to be ligated to remove the No.6i lymph nodes. Finally, the inferior wall of the duodenal bulb was skeletonized. Thus, the infrapyloric lymph nodes were removed en bloc with the stomach. Routine dissection of No.14v lymph nodes was unnecessary, unless metastatic No.6 lymph nodes were intraoperatively suspected. The operators need to avoid injuring the pancreas, especially when there was a tongue papillae of pancreas in this area, as well as the duodenum when approaching it.

### Statistical analysis

The SPSS 19.0 statistics software (SPSS Inc., Chicago, IL) was used to conduct all statistical analyses. Continuous data were expressed as mean ± standard deviation, and categorical variables were expressed as number (%). Statistical significance was defined as two-sided p < 0.05.

## Results

Totally, one hundred and eighteen patients (92 male, 78%) were included in analysis. The mean age was 55.4 ± 10.7 years and the body mass index was 22.2 ± 2.8 kg/m^2^. The tumor located in 74 patients at the lower 1/3 of the stomach, 11 at middle 1/3 and 33 at upper 1/3. The patients’ characteristics are summarized in Table 1.

**Table 1:** Clinicopathologic characteristics, operative and pathological results of the patients

Variables	N = 118 [Median, Range]
Demographics	
Male/female	92 (78.0%)/26 (22.0%)
Age (years)	55.4 ± 10.7 [30–77]
Body mass index (kg/m^2^)	22.2 ± 2.8 [22.1, 16–30.4]
Tumor location (Upper/Middle/Lower)	33 (28.0%)/11 (9.3%)/74 (62.7%)
Operative results	
Gastrectomy (Total/distal)	35 (29.7%)/83 (70.3%)
Operative time (minutes)	246.6 ± 45.7 [240, 175–440]
Time for No.6 lymphadenectomy (minutes)	32.1 ± 5.8 [25–42]
Blood loss (mL)	87.0 ± 35.9 [20–230]
Open conversion	0
Postoperative recovery	
Mortality	0
Complications	21 (17.8%)
Pulmonary infections or plural effusion	15 (12.7%)
Intraperitoneal abscesses	3 (2.5%)
Surgical site infections	1 (0.8%)
Gastroplegia	2 (1.7%)
First flatus (days)	4.9 ± 1.3 [2–8]
First liquid diet intake (days)	3.3 ± 1.1 [2–6]
Postoperative hospital stay (days)	9.6 ± 3.7 [6–28]
Pathologic results	
Tumor size (cm)	3.0 ± 1.4 [3, 0.5–8]
No. of total retrieved lymph nodes	36.8 ± 12.9 [12–79]
No. of retrieved No.6 lymph nodes	5.1 ± 3.1 [0–17]
Mean number of overall metastatic lymph nodes	1.8 ± 2.9 [0–14]
Mean number of No.6 metastatic lymph nodes	0.2 ± 0.6 [0–3]
Patients with No.6 lymph nodes metastasis	15 (12.7%)
Stage^*^ I/II/III	57 (48.3%)/36 (30.5%)/25 (21.2%)

Data are mean ± standard deviation or n (%)

*According to the 7th edition Cancer Staging, American Joint Committee on Cancer

The laparoscopic gastrectomy with D2 lymphadenectomy was successful without open conversion in all patients. Thirty five patients underwent total gastrectomy and 83 patients distal gastrectomy. The mean operation time was 246.6 ± 45.7 min, where the mean time for No.6 lymph nodes dissection was 32.1 ± 5.8 min. The mean estimated blood loss was 87.0 ± 35.9 mL.

There was no peri-operative mortality. Postoperative complications happened in 21 patients (17.8%), including pulmonary infections or plural effusion in 15, intraperitoneal abscesses in 3, surgical site infections in one, and gastroplegia in 2. All complications were successfully treated with conservative therapy or aspiration. There was no No.6 lymphadenectomy-associated complications, such as injury of transverse colon, blood vessels of mesocolon, pancreas or duodenum, pancreatitis, pancreatic leakage and postoperative hemorrhage. The mean time to first flatus was 4.9 ± 1.3 days, mean time to first food intake 3.3 ± 1.1 days and mean hospital stay 9.6 ± 3.7 days.

The mean tumor size was 3.0 ± 1.4 cm. The mean number of total lymph nodes harvested was 36.8 ± 12.9 and No.6 lymph nodes retrieved 5.1 ± 3.1, where the mean number of overall metastatic lymph nodes was 1.8 ± 2.9 and metastatic No.6 lymph nodes 0.2 ± 0.6. No.6 lymph nodes metastases were observed in 15 patients. There were 35 patients in stage Ia, 22 stage Ib, 23 stage IIa, 13 stage IIb, 16 stage IIIa, 6 stage IIIb and 3 stage IIIc according to the 7th AJCC Cancer Staging.

## Discussion

In the present study, we showed satisfactory short term outcomes and dissecting efficacy of laparoscopic infrapyloric lymphadenectomy for gastric cancer. Compared with our 53 patients receiving traditional procedure going inside the omental sac and dissecting the No.6 lymph nodes from the lower edge of pancreas toward to the duodenum, the novel approach retrieved more total lymph nodes (36.8 ± 12.9 vs. 31.8 ± 10.5, p = 0.009) and No.6 lymph nodes (5.1 ± 3.1 vs. 3.7 ± 2.1, p = 0.002). Meanwhile, the operation time (246.6 ± 45.7 min vs. 261.5 ± 52.6 min, p = 0.078), volume of blood loss (87.0 ± 35.9 mL vs. 93.8 ± 30.8 mL, p = 0.212), and postoperative complication rates (17.8% vs. 22.6%, p = 0.458) were comparable. No No.6 lymphadenectomy-associated complications were observed. Lastly, the mean number of total lymph nodes harvested and No.6 lymph nodes retrieved was comparable, or even higher than those of laparoscopic or open surgery reported in previous literatures [14, 19–23].

Lymphadenectomy is an important component of gastric cancer surgery, and D2 lymphadenectomy has been proven to decrease the locoregional recurrence and gastric cancer related deaths nowadays [7]. Because No.6 lymph nodes is one of the most frequently involved stations, complete No.6 lymph nodes dissection is crucial for a curative gastrectomy even in a D1 or D1 + lymphadenectomy for early cancer, let alone advanced cancer [6, 14]. No.6 lymph nodes mainly drain the lymphatic flow from the lower 1/3 of the stomach which remains the most common site of gastric cancer although the incidence of proximal tumor has been increasing in the past two decades [5, 24]. Therefore, the metastatic rate of No.6 lymph nodes is quite high. Our previous study found that the metastatic rate of No.6 lymph nodes was 28.1%, [14] and another study has reported that the No.6 lymph nodes were the most frequently affected lymph nodes with the positive rate of 34.3% among patients with distal gastric cancer [25]. Consequently, the likelihood of residual disease in infrapyloric lymph nodes might increase for gastric cancer patients receiving incomplete No.6 lymphadenectomy. For them, actually, the positive No.6 lymph nodes might not be removed completely. Nevertheless, a complete No.6 lymph nodes dissection during laparoscopic surgery should not be regarded as a technically easy procedure.

Bursectomy has been proposed by Japanese surgeons since 1960s with the purpose to eliminate the possible invisible tumor seeding in the lesser sac of peritoneal cavity and completely remove the infrapyloric lymph nodes [26, 27]. Unfortunately, bursectomy has not been demonstrated to provide better survival outcome for resectable gastric cancer than non-bursectomy in a multicenter randomized controlled trial [28]. However, bursectomy could yield more retrieved lymph nodes than non-bursectomy; [29] and Blouhos et al. considered that an easy and complete en bloc infrapyloric lymph nodes dissection could be achieved through the surgical plane of right-sided bursectomy [27]. Although retrieving more lymph nodes does not necessarily associate with better long-term clinical outcomes, the removal of more lymph nodes improves the chance of cure theoretically and decreases the possibility of stage migration. In the present study, we also showed that laparoscopic infrapyloric lymph nodes dissection through the right bursa omentalis approach could contribute defining the proper surgical plane, which could minimize potential free cancer cell leakage caused by the transection of lymphatic vessels in an inappropriate plane, [30, 31] and facilitate an easy and complete lymphadenectomy. Although bursectomy is a technically challenging procedure especially performed with laparoscopy, our previous study showed that it could be performed safely by experienced surgeons [29]. Also, no bursectomy-associated complications were observed in the present study. The major risks of laparoscopic No.6 lymph nodes dissection through the right bursa omentalis approach are potential iatrogenic injury of colonic vessels or pancreatic parenchyma. Therefore, a thorough understanding of the anatomy of this area is critical. Identification of important anatomic structures, including the marginal vessel of the transverse colon, the middle colic vessels, inferior border of the pancreas, is crucial in order to avoid iatrogenic injury and recognize the correct surgical planes. Additionally, the interfascial space is generally loose on the right aspect of the transverse mesocolon. Thus, it is easier to perform the right-sided bursectomy only.

**Fig. 3:**
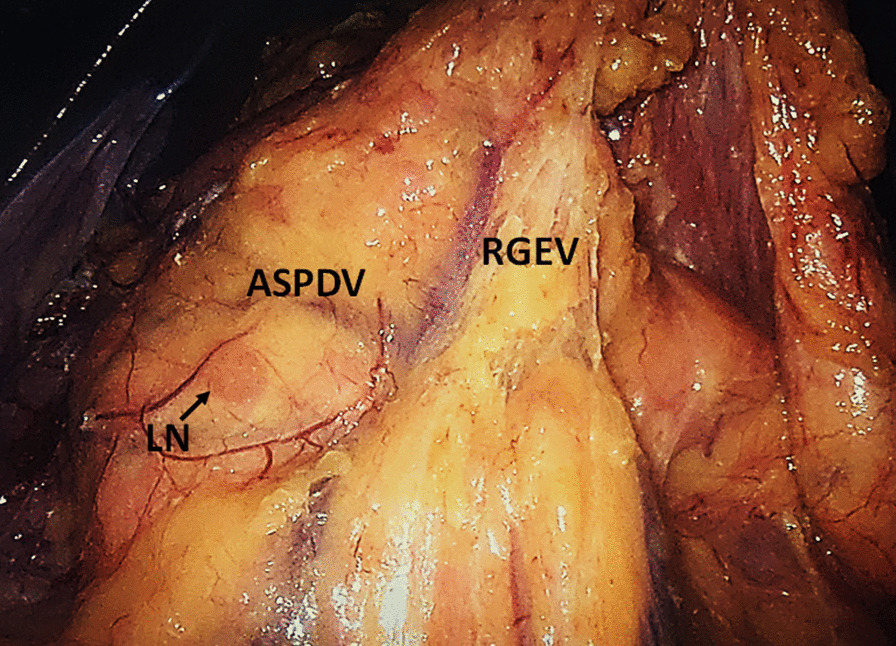
Lymph nodes (LN) located just below to the anterior superior pancreatoduodenal vein (ASPDV). *RGEV* right gastroepiploic vein

Our approach provides potential benefits for laparoscopic No.6 lymphadenectomy. Firstly, our procedure could provide a more complete infrapyloric lymph nodes dissection and avoid omission of lymph nodes, such as the right part of No.6v lymph nodes located near to the duodenum and the lymph nodes clinging to the pancreatic capsule, which is prone to be ignored when dissected in laparoscopic manner. Secondly, we indeed found lymph nodes locating just below the anterior superior pancreatoduodenal vein (Fig. 3), although the frequency was not high. Even though these lymph nodes should not be recognized as No.6 lymph nodes according to the Japanese Gastric Cancer Classification, [8] we believe that these lymph nodes have significant lymphatic channel that drains in the infrapyloric region, and communicate with the No.6 lymph nodes broadly. Therefore, we advocate to dissect these lymph nodes simultaneously and the right bursa omentalis approach is helpful to identify and dissect them. In addition, our procedure is also beneficial to expose the anterior superior pancreatoduodenal vein and the confluence of anterior superior pancreaticoduodenal vein to the right gastroepiploic vein. Finally, this procedure facilities defining the correct surgical plane and removing the whole greater omentum, which is usually integrated in the standard gastrectomy for T3 or deeper tumors [6]. A wrong plane might cause the residual of the greater omentum and residual No.6 lymph nodes at the side of the transverse mesocolon.

This study has a few limitations. Firstly, this study is a retrospective, single arm study. Nevertheless, as discussed above the current approach demonstrated comparable or better results compared with traditional procedure. Secondly, some patients with early stages had been included in the study. From our point of view, the loosed natural surgical plane is helpful to reduce the iatrogenic injury of blood vessels and adjacent organs, which facilitated an easier, safer and more complete infrapyloric lymph nodes dissection. It was reported that an easy and complete en bloc infrapyloric lymph nodes dissection could be achieved through the surgical plane of right-sided bursectomy [27]. However, one should notice that for patients with early stages, our procedure was a comparably more aggressive surgical approach, and cautions need to be warranted when performing the procedure on patients with early stages. Despite these limitations, the purpose of this study was to describe the laparoscopic infrapyloric lymph nodes dissection through the right bursa omentalis approach in detail and to present data suggest the safety and feasibility of this procedure. To our knowledge, this is the first study to describe a detailed procedure for performing laparoscopic infrapyloric lymph nodes dissection through the right bursa omentalis approach for gastric cancer.

## Conclusions

Laparoscopic infrapyloric lymph nodes dissection through the right bursa omentalis approach seems to be feasible and safe, facilitating a more complete No.6 lymphadenectomy for gastric cancer. The positive results need to be confirmed with prospective studies.

## Supplementary Information

**Additional file 1: Video S1.** This operation video shows the surgical technique of laparoscopic infrapyloric lymph nodes dissection through the right bursa omentalis approach.

## Data Availability

The datasets generated and/or analyzed during the study can be obtained from the corresponding Author on reasonable request.
